# Processes of change in trauma-focused CBT

**DOI:** 10.1080/20008198.2020.1866421

**Published:** 2021-02-01

**Authors:** Anke Ehlers, Milan Wiedemann, Hannah Murray, Esther Beierl, David M. Clark

**Affiliations:** Centre for Anxiety Disorders and Trauma, Department of Experimental Psychology, University of Oxford, Oxford, UK

## Abstract

**Background**: Numerous studies have shown that trauma-focused psychological treatments for PTSD are effective (Lewis, Roberts, Andrew, Starling, & Bisson, 2020). Few studies to date have investigated the processes of change during treatment. There is initial evidence that these treatments change problematic beliefs about the trauma and its consequences, and that belief change drives symptom change (Brown, Belli, Asnaani, & Foa, 2018). Further studies of other candidate processes are needed.

**Objective**: To investigate the temporal relationships between changes in theory-derived (Ehlers & Clark, 2000) treatment specific processes (appraisals, trauma memory characteristics, and unhelpful cognitive and behavioural responses) and non-specific processes (working alliance) and symptom change during a course of cognitive therapy for PTSD (e.g. Wild et al., 2020).

**Study 1 Methods**: Session by session changes in self-reports of cognitive factors and PTSD symptoms obtained from patients treated in routine clinical care were analysed with bivariate latent change score models (LCSM).

**Results**: Were preceded by changes in for most measures, changes in cognitive factors preceded changes in PTSD symptoms, but not vice versa.

**Study 2 Methods**: Patient and therapist ratings of working alliance were taken repeatedly. Autoregressive, cross-lagged models were used to examine whether working alliance predicted PTSD symptom severity at the next assessment point and vice versa.

**Results**: Higher patient and therapist working alliance was associated with lower PTSD symptoms. Crossed-lagged associations were found for therapist-rated alliance, but not for patient-rated alliance. A better working alliance at the start of treatment predicted treatment outcome. For most measures, changes in cognitive factors preceded changes in PTSD symptoms, but not vice versa.

**Conclusions**: Study 1 extended previous findings that that cognitive and behavioural processes suggested by theoretical models of PTSD play a key role in driving symptom improvement during trauma-focused CBT. Changes in cognitive factors preceded symptom change. This result suggests that monitoring these processes during therapy may help therapist in maximising treatment outcomes in individual patients, and may also point to further ways of optimising treatments in general. Study 2 suggested that working alliance may be an important factor in setting the necessary conditions for effective treatment, but did not find evidence of a temporal precedence of changes in working alliance driving symptom change.

## References

[cit0001] Brown, L. A., Belli, G. M., Asnaani, A., & Foa, E. B. (2018). A review of the role of negative cognitions about oneself, others, and the world in the treatment of PTSD. *Cognitive Therapy and Research*, 43(143–173). doi:10.1007/s10608-018-9938-1

[cit0002] Ehlers, A., & Clark, D. M. (2000). A cognitive model of posttraumatic stress disorder. *Behaviour Research and Therapy*, 38(4), 1–1. doi: 10.1016/S0005-7967(99)00123-0 10761279

[cit0003] Lewis, C., Roberts, N. P., Andrew, M., Starling, E., & Bisson, J. I. (2020). Psychological therapies for post-traumatic stress disorder in adults: Systematic review and meta-analysis. *European Journal of Psychotraumatology*, 11, (1). 10.1080/20008198.2020.1729633 PMC7144187 32284821

[cit0004] Wild, J., Warnock-Parkes, E., Murray, H., Kerr, A., Thew, G., Grey, N., … Ehlers, A. (2020). Treating posttraumatic stress disorder remotely with cognitive therapy for PTSD. *European Journal of Psychotraumatology*, 11, (1). 10.1080/20008198.2020.1785818 PMC7473124 33029325

